# Comprehensive Characterization of COVID-19 Patients with Repeatedly Positive SARS-CoV-2 Tests Using a Large U.S. Electronic Health Record Database

**DOI:** 10.1128/spectrum.00327-21

**Published:** 2021-08-18

**Authors:** Xiao Dong, Yujia Zhou, Xiao-ou Shu, Elmer V. Bernstam, Rebecca Stern, David M. Aronoff, Hua Xu, Loren Lipworth

**Affiliations:** a School of Biomedical Informatics, The University of Texas Health Science Center at Houston, Houston, Texas, USA; b Division of Epidemiology, Department of Medicine, Vanderbilt University Medical Center, Nashville, Tennessee, USA; c Division of General Internal Medicine, Department of Internal Medicine, McGovern Medical School, The University of Texas Health Science Center at Houston, Houston, Texas, USA; d Division of Infectious Diseases, Department of Medicine, Vanderbilt University Medical Center, Nashville, Tennessee, USA; Memorial Sloan Kettering Cancer Center

**Keywords:** COVID-19, EHR, reinfection

## Abstract

In the absence of genome sequencing, two positive molecular tests for severe acute respiratory syndrome coronavirus 2 (SARS-CoV-2) separated by negative tests, prolonged time, and symptom resolution remain the best surrogate measure of possible reinfection. Using a large electronic health record database, we characterized clinical and testing data for 23 patients with repeatedly positive SARS-CoV-2 PCR test results ≥60 days apart, separated by ≥2 consecutive negative test results. The prevalence of chronic medical conditions, symptoms, and severe outcomes related to coronavirus disease 19 (COVID-19) illness were ascertained. The median age of patients was 64.5 years, 40% were Black, and 39% were female. A total of 83% smoked within the prior year, 61% were overweight/obese, 83% had immunocompromising conditions, and 96% had ≥2 comorbidities. The median interval between the two positive tests was 77 days. Among the 19 patients with 60 to 89 days between positive tests, 17 (89%) exhibited symptoms or clinical manifestations consistent with COVID-19 at the time of the second positive test and 14 (74%) were hospitalized at the second positive test. Of the four patients with ≥90 days between two positive tests (patient 2 [PT2], PT8, PT14, and PT19), two had mild or no symptoms at the second positive test and one, an immunocompromised patient, had a brief hospitalization at the first diagnosis, followed by intensive care unit (ICU) admission at the second diagnosis 3 months later. Our study demonstrated a high prevalence of compromised immune systems, comorbidities, obesity, and smoking among patients with repeatedly positive SARS-CoV-2 tests. Despite limitations, including a lack of semiquantitative estimates of viral load, these data may help prioritize suspected cases of reinfection for investigation and continued surveillance.

**IMPORTANCE** The comprehensive characterization of severe acute respiratory syndrome coronavirus 2 (SARS-CoV-2) testing and clinical data for patients with repeatedly positive SARS-CoV-2 tests can help prioritize suspected cases of reinfection for investigation in the absence of genome sequencing data and for continued surveillance of the potential long-term health consequences of SARS-CoV-2 infection.

## INTRODUCTION

As of 9 August 2021, there have been more than 202 million confirmed cases of coronavirus disease 19 (COVID-19) globally, including 35 million in the United States. A reverse transcriptase PCR (RT-PCR) test is considered the gold standard for detection of severe acute respiratory syndrome coronavirus 2 (SARS-CoV-2) in upper and lower respiratory specimens and for diagnosis of COVID-19. While neutralizing antibodies are detectable for several months following recovery from SARS-CoV-2 infection (1, 2), it remains unknown whether and for how long these antibody responses protect patients from reinfection. There have been many case reports of patients with a second positive PCR test after their PCR results turned negative and symptoms resolved (3). Most of these reports are suspected cases of reinfection based on limited clinical or testing data; in a minority of suspected cases of reinfection, the viral genome sequences were analyzed and shown to be distinct, strongly supporting a reinfection rather than failure to clear an initial infection (4). In the absence of genomic evaluations, the presence of two positive molecular tests separated by negative tests, prolonged time, and clinical resolution of symptoms remain the best surrogate measure of possible reinfection. Using the Centers for Disease Control and Prevention Common Investigation Protocol for Investigating Suspected SARS-CoV-2 Reinfection (4) as a guide, we conducted a comprehensive evaluation of patients who had repeated positive SARS-CoV-2 PCR tests in a large U.S. COVID-19 electronic health record (EHR) database. We characterized their demographic and clinical characteristics, including their SARS-CoV-2 testing journey, symptoms, medication use, and COVID-19-related complications.

## RESULTS

**FIG 1:**
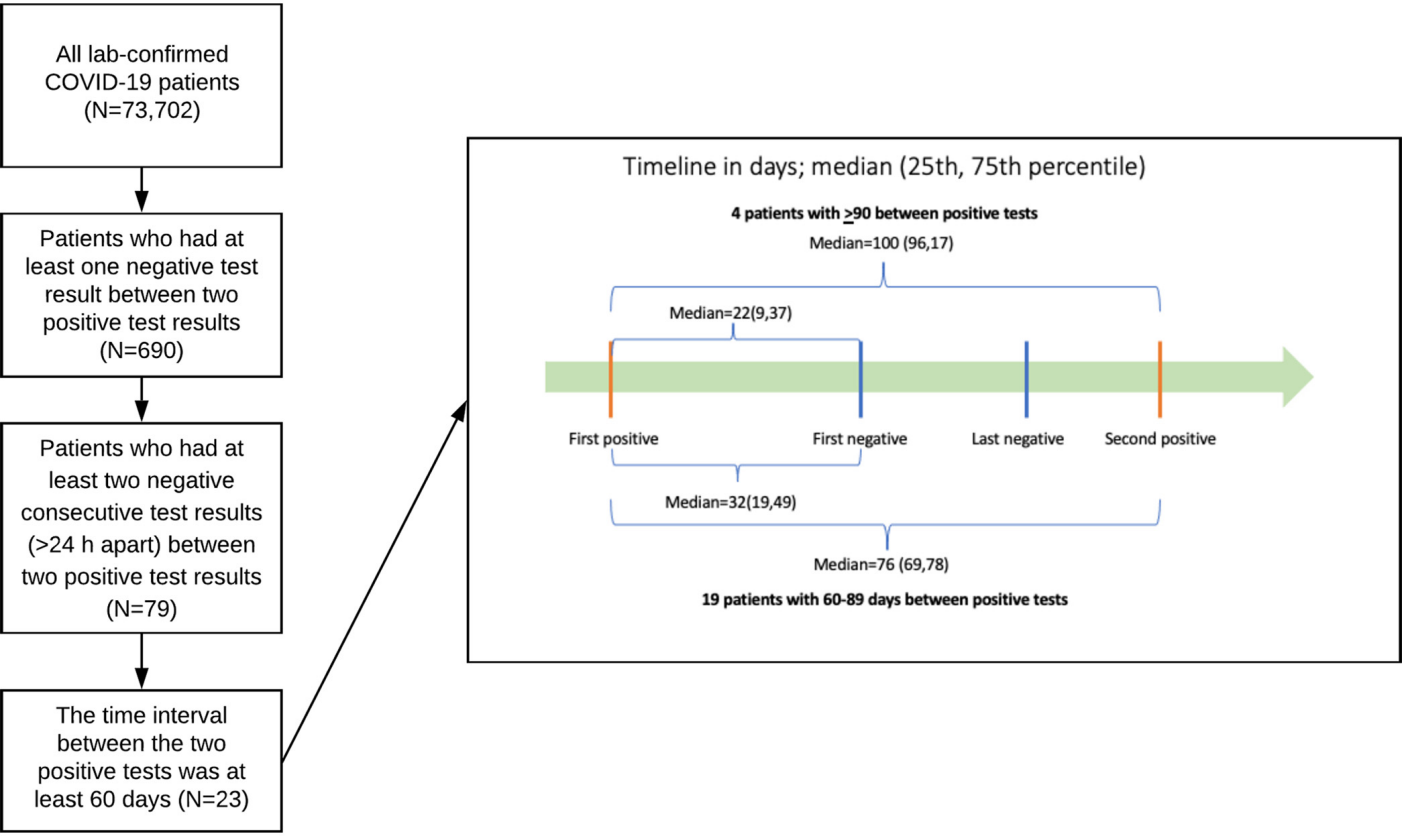
Study flow chart and SARS-CoV-2 PCR testing timeline (days) for 23 repeatedly positive patients.

For the 4 patients with at least 90 days between positive tests, the median interval between the 2 positive tests, separated by 2 or more consecutive negative tests >24 h apart, was 100 days (25th, 75th percentile of 96, 107), and the median interval between the first positive and first negative test was 22 days (9, 37) (Fig. 1). For the 19 patients with 60 to 89 days between positive tests, the corresponding intervals were 76 days (69, 78) and 32 days (19, 49), respectively (Fig. 1).

The median age of the 23 repeatedly positive patients at the index date was 64.5 years (25th, 75th percentile of 53.5, 69.8). Seventeen patients were diagnosed in the Northeast, 5 in the Midwest, and 1 in the South; 40% of patients were Black, 40% white, and 20% other/unknown race; 83% had non-Hispanic ethnicity; and 39% were female. Almost 83% of patients smoked within the prior year, and 61% were overweight or obese.

**FIG 2:**
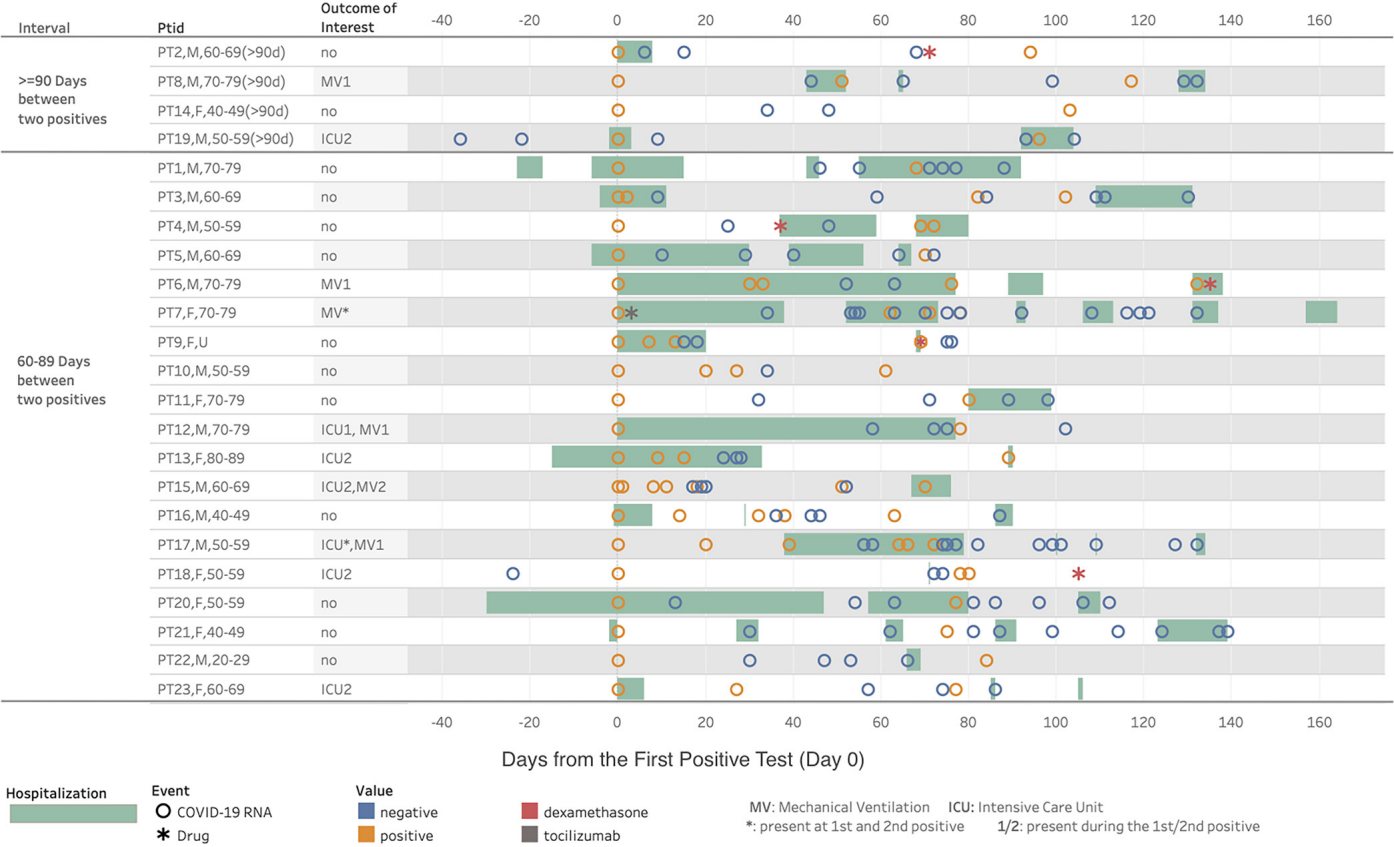
COVID-19 RT-PCR testing and clinical journey for 23 patients with repeatedly positive tests.

Comorbidity diagnoses and symptom prevalence for the 23 individual patients at the time of each positive test are presented in Table 1, and their PCR test and clinical journeys are shown in Fig. 2. Chronic disease prevalence was high, including hypertension (70%), CVD, atrial fibrillation or CKD (each 26%), and insulin-dependent type 2 diabetes or history of venous thromboembolism/long-term anticoagulation (each 22%). Overall, 96% of patients had ≥2 comorbidities. Most notably, 19 of the patients (83%) had immunocompromising conditions, including 2 of the 4 patients with ≥90 days between positive tests (patient 14 [PT14] and PT19).

**TABLE 1:** Individual-level comorbidity and symptom data for the 23 repeatedly positive patients^*a*^

Days between positive SARS-CoV-2 tests	Patient	Comorbidities^*b*^	Symptom(s) of COVID-19 episode 1	Symptom(s) of COVID-19 episode 2
≥90	2	HTN, HLD	SOB, cough, fever, chest pain, pneumonia, acute respiratory failure w/hypoxia, bradycardia	None
	8	Nicotine, AFib, HTN, Pacemaker, Long QT, h/o VTE, long-term AC	None	SOB, bradycardia
	14	Nicotine, **HIV**, **alcoholic cirrhosis**, **protein-calorie malnutrition**, **alcohol dependence**, **TB**	Low back pain	Fever
	19	HTN, **alcohol dependence**	Fever, pneumonia, hypoxia, AKI	Fever, respiratory failure w/hypoxia, encephalopathy, AKI
60–89	1	Insulin-dependent DM2 w/CKD, COPD, nicotine, AFib, HTN, HLD, NSTEMI, pacemaker, long QT, h/o VTE, long-term AC, **ESRD on HD**, **retroperitoneum cancer**	SOB, diarrhea, weakness, low back pain, pneumonia, acute respiratory failure w/hypoxia, ARDS, altered mental status, metabolic encephalopathy, fluid overload, ventricular tachycardia	Acute respiratory failure w/hypoxia, ventricular tachycardia
	3	**Kidney-heart transplant**, **rheumatoid arthritis**, **protein-calorie malnutrition**	Cough, chest pain, pneumonia, AKI, tachycardia	SOB, diarrhea, respiratory failure w/hypoxia, AKI, tachycardia
	4	HTN, HLD, **HIV**, **alcoholic cirrhosis** w/ascites	Fever, tachycardia	SOB, fever, AKI, tachycardia, acute embolism and thrombosis
	5	**Prostate cancer**, **alcoholic cirrhosis** w/ascites, **alcohol abuse**, **h/o pulmonary TB**	Cough, fever, headache, chest pain, tachycardia, acute embolism and thrombosis	Chest pain, tachycardia, acute embolism and thrombosis
	6	HTN, HLD, old MI, NSTEMI, long QT, **ESRD on HD**, **protein-calorie malnutrition**, OSA, **thyroid cancer**	Fever, diarrhea, pneumonia, acute respiratory failure w/hypoxia, ARDS, ventilator dependence, AKI, encephalopathy, tachycardia, severe sepsis w/shock, acute embolism and thrombosis	Diarrhea, pneumonia, acute respiratory failure w/hypoxia, ARDS, AKI, tachycardia, sepsis w/shock, acute embolism and thrombosis
	7	Insulin-dependent DM2 w/CKD, AFib, HTN, HLD, long QT, long-term AC, CKD, **kidney transplant**, **protein-calorie malnutrition**	SOB, cough, headache, pneumonia, acute respiratory failure with hypoxia, ARDS, AKI, encephalopathy, fluid overload, sepsis w/shock, acute embolism and thrombosis	Fever, pneumonia, acute respiratory failure w/hypoxia, ventilator dependence, AKI, fluid overload, sepsis w/shock
	9	AFib, HTN, HLD, old MI, pacemaker, long QT, long-term AC, CKD, **breast cancer**, **protein-calorie malnutrition**	Weakness, pneumonia, acute respiratory failure w/hypoxia, AKI	Diarrhea, weakness, pneumonia, acute respiratory failure w/hypoxia, AKI
	10	HTN, **CLL w/o remission**	SOB, fever, headache	None
	11	Insulin-dependent DM2, nicotine, HTN, HLD, old MI, **cirrhosis**	Altered mental status	Weakness, AKI, metabolic encephalopathy, altered mental status, bradycardia
	12	AFib, HTN, HLD, CKD, **protein-calorie malnutrition**	SOB, diarrhea, pneumonia, acute respiratory failure w/hypoxia, ARDS, AKI, encephalopathy, tachycardia, sepsis w/shock	SOB, diarrhea, chest pain, pneumonia, acute respiratory failure w/hypoxia, ARDS, AKI, encephalopathy, tachycardia, sepsis w/shock
	13	AFib, HTN, HLD, long-term AC, **cirrhosis**, **protein-calorie malnutrition**	Pneumonia, acute respiratory failure w/hypoxia, tachycardia	Diarrhea, chest pain, pneumonia, acute respiratory failure w/hypoxia, AKI, encephalopathy, sepsis w/shock
	15	Insulin-dependent DM2 w/CKD, HTN, HLD, old MI, **ESRD on HD**, heart transplant	Fever, headache, diarrhea, weakness, low back pain, pneumonia, acute respiratory failure w/hypoxia, metabolic encephalopathy, fluid overload	Weakness, pneumonia, acute respiratory failure w/hypoxia, encephalopathy, fluid overload, tachycardia, sepsis w/o shock
	16	HTN, HLD	Weakness, pneumonia, AKI, metabolic encephalopathy	Fever, weakness, chest pain, pneumonia, tachycardia
	17	HTN, protein-calorie malnutrition	Pneumonia, acute respiratory failure w/hypoxia, AKI, encephalopathy	SOB, pneumonia
	18	**Esophageal cancer**	None	None
	20	Insulin-dependent DM2 w/CKD, nicotine, HTN, ESRD on HD, HIV	Fever, headache, chest pain, acute respiratory failure w/hypoxia	Chest pain
	21	Nicotine, long QT	Low back pain	Low back pain
	22	**Monoclonal gammopathy**, **respiratory TB**, **histoplasmosis**, **blastomycosis**	None	Fever
	23	COPD, nicotine	Pneumonia	SOB, pneumonia, acute respiratory failure w/hypoxia

a Data are based on manual review of ICD-10 codes within 30 days before and after the index date and the second positive test date. MI, myocardial infarction; AC, anticoagulation; HTN, hypertension; HDL, hyperlipidemia; SOB, shortness of breath; DM2, type 2 diabetes mellitus; CKD, chronic kidney disease; AFib, atrial fibrillation; VTE, venous thromboembolism; ESRD, end-stage renal disease; HD, hemodialysis; TB, tuberculosis; OSA, obstructive sleep apnea; AKI, acute kidney injury; ARDS, acute respiratory distress syndrome; CLL, chronic lymphocytic leukemia; COPD, chronic obstructive pulmonary disease.

b Bold text denotes an immunocompromising condition.

For individuals with 45 to 89 days between positive SARS-CoV-2 tests, CDC investigative criteria included having “a symptomatic second episode and no obvious alternate etiology for COVID-19–like symptoms OR close contact with a person known to have laboratory-confirmed COVID-19.” Among the 19 patients in our study with 60 to 89 days between positive tests, 17 (89%) exhibited symptoms or clinical manifestations consistent with COVID-19 at the time of the second positive test, including 9 (47%) with acute respiratory failure, 8 (42%) with acute kidney failure, 6 (32%) with shortness of breath, 5 (26%) with fever, and 3 (16%) with acute embolism and thrombosis. Fourteen of the 19 (74%) patients were hospitalized at the second positive test, of whom all but 4 were also hospitalized at the first positive SARS-CoV-2 test. One patient was treated with tocilizumab (PT7, at the time of first positive test and during an extended hospitalization) and 4 were treated with dexamethasone after the first diagnosis of COVID-19.

As shown in Fig. 2 and Table 1, four of the patients (PT5, PT7, PT12, and PT17) with immunocompromising conditions had severe symptoms and lengthy hospitalizations (including ICU and mechanical ventilation for PT12 and PT17) beginning at the first positive COVID-19 test and numerous negative tests, often with their second positive test in close proximity to one or multiple negative tests. Additionally, PT10 had no COVID-19-like symptoms or related treatments at the second positive test, and PT18 had esophageal cancer and no COVID-19-like symptoms at the time of either positive test. Neither PT5 nor PT10 was assigned an ICD-10 code for COVID-19 (ICD10 U07.1) at the time of the second positive SARS-CoV-2 test. The clinical journeys of these six repeatedly positive patients cast doubt about the accuracy of categorizing them as true reinfections.

Of the four patients (Fig. 2, top) who had ≥90 days between two positive tests (PT2, PT8, PT14, and PT19), the record of one immunocompromised patient (PT14) suggests mild-to-moderate disease with few symptoms following both COVID-19 diagnoses. PT19, who was also immunocompromised, had a brief hospitalization at the first diagnosis, followed by ICU admission at the time of the second positive test 3 months later. PT2 had severe symptoms, hospitalization, and treatment with dexamethasone after the first positive test, but no symptoms or treatment at the second positive test. Notably, neither PT2 nor PT19 was assigned an ICD-10 code for COVID-19 (ICD10 U07.1) at the time of the second positive SARS-CoV-2 test. Patient 8 had a pacemaker and two hospitalizations, and the symptoms noted were shortness of breath and bradycardia at the second positive test; it is unclear why this patient was initially tested for COVID-19 in the absence of symptoms or a noted preoperative examination.

No patients had cardiac arrest, tracheostomy, amputation, or death.

## DISCUSSION

This study provides clinical and testing characterization of 23 COVID-19 patients with suspected reinfection, defined as repeatedly positive SARS-CoV-2 PCR tests separated by consecutive negative tests and prolonged time. Over one-third of patients were of Black race, and we observed a high prevalence of obesity and multiple comorbidities known to increase the risk of COVID-19 illness, including hypertension, diabetes, and CKD. Moreover, 83% of the patients with repeated positivity were current smokers, which is linked to an increased risk of severe COVID-19 (5). It is possible that those known to be at a particularly high risk for COVID-19 or those with persistent or recurrent symptoms may undergo frequent testing, thereby increasing the likelihood of receiving some false-positive or false-negative results.

Immunocompromising conditions, including end-stage renal disease on dialysis; HIV; cirrhosis, including alcohol-related; solid organ transplant; cancer; and protein-calorie malnutrition, were more common in our study population affected by COVID-19 than in the general population without COVID-19. Consistent with prior evidence that immunocompromised populations with COVID-19 have elevated risks of COVID-19 severity and morbidity and mortality (6–9), among the subset of patients in our study with immunocompromising conditions, more than two-thirds required hospitalization for the second positive PCR test after the interval negative PCR test. Patients with HIV in our sample had at least one other significant comorbidity, namely, either alcoholic cirrhosis (PT4 and PT14) or ESRD on hemodialysis (PT20). Reinfection may therefore raise clinical suspicion for an underlying immune defect, which may have also influenced the duration to achieve viral clearance.

Over 90% of patients in our study exhibited symptoms or clinical manifestations consistent with COVID-19 at the time of the second positive test, satisfying a CDC criterion for the investigation of suspected SARS-CoV-2 reinfection, particularly among those with 45 to 89 days between positive PCR tests. However, a biased detection of symptomatic reinfection is possible (10), and some of the symptoms could be related to the multitude of other underlying chronic medical conditions present among these patients, while the second positive COVID PCR test could be due to prolonged shedding or a false positive. Overall, 70% (12/17) of patients hospitalized at the first positive test were also hospitalized at the second test, suggesting that in many cases reinfection was not associated with less severe disease in our study population. In a recent review of 16 reported cases of reinfection confirmed by sequencing (10), for those 12 cases in whom severity could be compared between episodes, one-half of reinfections were less severe, which the authors suggested may reflect partial immune protection. However, the demographics of that study population differed from ours, as 8 of the 16 cases were between the ages of 20 to 30 years and 7 were health care workers, with a high potential for reinfection.

Overall, in our study, 37% of those hospitalized had severe disease characterized by ICU admission. This value is higher than previous estimates that 17% to 35% of hospitalized COVID-19 patients are treated in an ICU (2). Acute kidney injury (AKI) has been reported to occur in approximately 9% of hospitalized COVID-19 patients and a higher proportion of those requiring ICU admission (2). We observed AKI as a more common complication associated with COVID-19, including after both COVID-19 diagnoses in several individuals, but we were unable to determine whether they were independent or persistent events.

It is possible that hospitalized patients are more likely to undergo frequent testing, due to more severe disease or to support discharge to a rehabilitation facility or nursing home. This frequent testing can lead to alternating positive and negative tests, often on overlapping days. Given the high hospitalization rate in our study, repeated positive tests for some patients (e.g., PT5, PT7, PT12, and PT17) occurring during the time of an extended hospitalization with severe complications may not represent true reinfections. Moreover, prolonged viral shedding, as has been observed in severe COVID-19 cases (11), cannot be ruled out. A recent analysis in the Emory Healthcare System indicated that, among 22,443 patients who had at least 2 tests, the median (interquartile range [IQR]) duration between first and last positive test was 19 days (12, 32), and a duration of 45 and 90 days represented the 88th and 97th percentile, respectively (10).

In the absence of genomic evaluations to definitively confirm reinfection (3, 10), finding two positive molecular tests separated by negative tests, prolonged time, and resolution of symptoms remain the best surrogate measure of possible reinfection. In 169 previously reported cases to date (3), with an average of 115 days between first and second positive test, viral genome sequences were shown to be distinct, strongly suggesting a reinfection rather than a failure to clear an initial infection. Our identification and clinical characterization of 23 possible reinfections in a large data set, with a median of 77 days between positive tests, provides additional data suggesting that reinfections may be common. Since most patients in the Optum data set did not have repeated tests after their COVID-19 diagnosis, the true incidence rate of recurrent detectable SARS-CoV-2 cannot be estimated.

Our analysis was limited by a lack of information on RT-PCR platforms or semiquantitative RT-PCR cycle threshold (*C_T_*) values. As additional assays became available during the course of the pandemic, various test sensitivities may have contributed to testing discrepancies during our study period. The patients in our study nevertheless fulfilled CDC criteria for cases ≥90 days apart or 45 to 89 days apart based on positive RT-PCR, and based on our study definition, cases were classified as reinfection rather than relapse based on interval negative RT-PCR. Genomic sequencing of samples would have allowed for a direct assessment of whether the CDC investigative criteria matched the genomic data and thus lend further credibility to the recommendations. We were not able to confirm if COVID-19 was the primary diagnosis prompting hospitalization, if patients were incidentally found to test positive for SARS-CoV-2 upon admission for an unrelated illness, or later became symptomatic during the hospitalization course. Diagnoses may be more likely to be incidental if associated with ICD-10 codes for preprocedural exam (e.g., elective surgery); however, a preprocedural exam at the time of the second positive test was noted for only one patient in our study (PT2). Finally, repeatedly positive tests do not necessarily mean a reinfection, and persistent infection or relapse cannot be ruled out, particularly if signs and symptoms observed at the second positive test are similar to those seen in individuals with post-acute sequelae of COVID-19 (or “long COVID”).

Despite these limitations, our study provides a comprehensive characterization of demographic, clinical, and SARS-CoV-2 testing data for patients with repeatedly positive SARS-CoV-2 tests in a large EHR database across the United States, which could help prioritize suspected cases of reinfection for investigation in the absence of sequencing data and for continued surveillance of potential long-term health consequences of SARS-CoV-2 infection. Further investigation into the risk of reinfection by type and degree of immunosuppressive condition, medications, and disease chronicity, as well as evaluation of immune response after initial SARS-CoV-2 infection, will be valuable for future goals of prevention, mitigation of risk factors, and reduction of illness severity.

## MATERIALS AND METHODS

This retrospective study used the COVID-19 data set collected by Optum (12), a unit of United Health Group that provides multiple services, including health plans, pharmacy benefits, data, and analytics. The data set implements a low-latency data acquisition model that aggregates deidentified EHR data from over 700 hospitals and 7,000 clinics across the continuum of care. As of 20 August 2020, the Optum COVID-19 data set included 73,702 patients with a COVID-19 diagnosis code (ICD10 U07.1) that was laboratory confirmed with a positive SARS-CoV-2 PCR test, of whom 690 had 2 positive PCR test results separated by at least 1 negative test result. The study sample was further restricted to patients who had 2 consecutive negative test results >24 h apart between 2 positive test results (n = 79); of these patients, 4 had at least 90 days between their 2 positive tests and another 19 had at least 60 days between their 2 positive tests and had accessible demographic and clinical data (Fig. 1). If a negative test and a positive test were returned on the same day (<24 h), both tests were disregarded.

Demographic and clinical information, including age, gender, race/ethnicity, smoking status, and body mass index (BMI), was extracted. Smoking and BMI were based on the patient’s most recent record within 1 year prior to the index date (first SARS-CoV-2-positive test date). Available EHR data for the 23 patients were manually reviewed. The prevalence of chronic medical conditions which are considered risk factors for COVID-19 was ascertained, including insulin-dependent type 2 diabetes, hypertension, chronic kidney disease (CKD), respiratory disease (including chronic obstructive pulmonary disease), cardiovascular disease (CVD), atrial fibrillation, and immunocompromising conditions (including end-stage renal disease (ESRD) on dialysis; human immunodeficiency virus (HIV); cirrhosis, including alcohol-related; solid organ transplant; cancer; and protein-calorie malnutrition). Symptoms typical of COVID-19 were ascertained for each patient during each of two time periods, within 30 days before and after the index date and the second positive test date. In addition, severe clinical outcomes related to COVID-19 illness and medications commonly used to treat COVID-19 were ascertained during each time period, including the following: hospitalization, intensive care unit (ICU) admission, mechanical ventilation, tracheostomy, amputation, or death (at second positive test).

Continuous variables were expressed as median (25th and 75th percentile) and categorical variables as counts (percentages). Missing data were not imputed.
